# Supplementary material for: Trajectories of Lipid Profile and Risk of Carotid Atherosclerosis Progression: A Longitudinal Cohort Study
Source: Nutrients. 2022 Aug 8;14(15):3243. doi: 10.3390/nu14153243 (PMC9370402; doi:10.3390/nu14153243)
Supplement: Supplementary file 1 [file nutrients-14-03243-s001.zip › nutrients-1837015-supplementary.pdf]

Supplementary Table S1. Association between CAS progression and lipid trajectory classes from baseline in participants aged ≤ 65 years (*n* = 8657)

**Baseline lipid profiles & longitudinal trajectory**		**No. of Patients**	**CAS progression**	**HR (95%CI)**	***p* value**
TC	TC < 5.18 mmol/L	5599	961	Reference	
	TC ≥ 5.18 mmol/L & U-shape/inverse U-shape class	343	57	1.075 (0.822-1.406)	0.599
	TC ≥ 5.18 mmol/L& moderate-stable class	2667	606	1.458 (1.317-1.615)	0.000
TG	TG < 1.70 mmol/L & low-stable class	5339	944	Reference	
	TG < 1.70 mmol/L & moderate-stable/elevated-increasing class	350	76	1.152 (0.912-1.456)	0.235
	TG ≥ 1.70 mmol/L & low-stable class	605	109	1.086 (0.891-1.325)	0.413
	TG ≥ 1.70 mmol/L & elevated-increasing class	290	62	1.422 (1.100-1.839)	0.007
	TG ≥ 1.70 mmol/L & moderate-stable class	2025	433	1.247 (1.113-1.397)	0.000
LDL-C	LDL-C < 3.36 mmol/L	5553	948	Reference	
	LDL-C ≥ 3.36 mmol/L& U-shape class	241	42	1.326 (0.973-1.808)	0.074
	LDL-C ≥ 3.36 mmol/L& inverse U-shape class	89	20	1.102 (0.706-1.720)	0.669
	LDL-C ≥ 3.36 mmol/L& stable class	2726	614	1.523 (1.375-1.686)	0.000
HDL-C	HDL-C ≥ 1.04 mmol/L & inverse U-shape class	286	63	Reference	
	HDL-C ≥ 1.04 mmol/L & U-shape class	1041	170	0.852 (0.638-1.137)	0.276
	HDL-C ≥ 1.04 mmol/L & stable class	5602	1024	0.884 (0.686-1.141)	0.344
	HDL-C < 1.04 mmol/L	1680	367	1.008 (0.772-1.317)	0.953

Abbreviations: CAS, carotid atherosclerosis progression; HR, hazard ratio; CI, confidence interval; TC, total cholesterol; TG, Triglyceride; LDL-C, low-density lipoprotein cholesterol; HDL-C, high-density lipoprotein cholesterol.

Supplementary Table S2. Association between CAS progression and lipid trajectory classes from baseline in participants without chronic diseases (n = 8654)

Baseline lipid profiles & longitudinal trajectory		No. of Patients	CAS progression	HR (95%CI)	*p* value
TC	TC < 5.18 mmol/L	5555	900	Reference	
	TC ≥ 5.18 mmol/L & U-shape/inverse U-shape class	325	54	1.091 (0.828-1.438)	0.536
	TC ≥ 5.18 mmol/L& moderate-stable class	2634	573	1.438 (1.295-1.598)	0.000
TG	TG < 1.70 mmol/L & low-stable class	5500	922	Reference	
	TG < 1.70 mmol/L & moderate-stable/elevated-increasing class	326	66	1.139 (0.887-1.462)	0.307
	TG ≥ 1.70 mmol/L & low-stable class	579	98	1.029 (0.835-1.267)	0.790
	TG ≥ 1.70 mmol/L & elevated-increasing class	223	44	1.342 (0.992-1.816)	0.057
	TG ≥ 1.70 mmol/L & moderate-stable class	1886	397	1.248 (1.109-1.404)	0.000
LDL-C	LDL-C < 3.36 mmol/L	5547	895	Reference	
	LDL-C ≥ 3.36 mmol/L& U-shape class	215	39	1.357 (0.984-1.871)	0.644
	LDL-C ≥ 3.36 mmol/L& inverse U-shape class	92	19	1.114 (0.705-1.759)	0.063
	LDL-C ≥ 3.36 mmol/L& stable class	2660	574	1.527 (1.374-1.696)	0.000
HDL-C	HDL-C ≥ 1.04 mmol/L & inverse U-shape class	303	64	Reference	
	HDL-C ≥ 1.04 mmol/L & U-shape class	1110	175	0.879 (0.660-1.170)	0.377
	HDL-C ≥ 1.04 mmol/L & stable class	5552	971	0.845 (0.656-1.088)	0.192
	HDL-C < 1.04 mmol/L	1549	317	0.935 (0.715-1.224)	0.626

Chronic diseases were identified as hypertension and diabetes; Abbreviations: CAS, carotid atherosclerosis progression; HR, hazard ratio; TC, total cholesterol; TG, Triglyceride; LDL-C, low-density lipoprotein cholesterol; HDL-C, high-density lipoprotein cholesterol.

Supplementary Table S3. Hazard ratios (95% confidence intervals) of CAS progression by age-stratified baseline level and trajectory class of each lipid profile.

**Baseline lipid profiles & longitudinal trajectory**	***n***	**CAS progression**	**Unadjusted**		**Model 1**		**Model 2**	
			**Crude HR (95%CI)**	***p* value**	**Adjusted HR (95%CI)**	***p* value**	**Adjusted HR (95%CI)**	***p* value**
**Age ≤ 45 years**	3681	493						
TC								
Class 1: Moderate-stable	3620	485	Reference		Reference		Reference	
Class 2: Inverse U-shape	40	4	0.737 (0.275-1.974)	0.544	0.648 (0.242-1.735)	0.388	0.469 (0.150-1.461)	0.191
Class 3: U-shape	21	4	**3.027 (1.129-8.117)**	**0.028**	2.548 (0.950-6.835)	0.063	2.297 (0.852-6.191)	0.100
TG								
Class 1: Low-stable	2741	317	Reference		Reference		Reference	
Class 2: Elevated-increasing	108	23	**2.125 (1.387-3.254)**	**0.001**	1.309 (0.850-2.017)	0.222	1.200 (0.770-1.870)	0.420
Class 3: Moderate-stable	832	153	**1.593 (1.313-1.932)**	**0.000**	1.090 (0.891-1.334)	0.401	0.975 (0.787-1.208)	0.816
LDL-C								
Class 1: Stable	3616	483	Reference		Reference		Reference	
Class 2: U-shape	36	6	2.083 (0.929-4.672)	0.075	1.654 (0.738-3.711)	0.222	1.552 (0.691-3.488)	0.287
Class 3: Inverse U-shape	29	4	1.069 (0.399-2.861)	0.894	0.831 (0.310-2.224)	0.712	0.770 (0.287-2.064)	0.603
HDL-C								
Class 1: Stable	3205	450	Reference		Reference		Reference	
Class 2: Inverse U-shape	111	10	0.621 (0.332-1.163)	0.137	0.719 (0.384-1.346)	0.302	0.708 (0.378-1.328)	0.282
Class 3: U-shape	365	33	0.708 (0.497-1.009)	0.056	0.990 (0.688-1.423)	0.955	1.141 (0.783-1.662)	0.493
**Age 45-65 years**	4961	1136						
TC								
Class 1: Moderate-stable	4646	1079	Reference		Reference		Reference	

Class 2: Inverse U-shape	139	32	0.781 (0.549-1.112)	0.170	0.806 (0.566-1.148)	0.233	0.829 (0.582-1.182)	0.300
Class 3: U-shape	176	25	**0.632 (0.425-0.940)**	**0.024**	**0.665 (0.446-0.989)**	**0.044**	**0.657 (0.433-0.995)**	**0.047**
TG								
Class 1: Low-stable	3229	741	Reference		Reference		Reference	
Class 2: Elevated-increasing	198	42	1.037 (0.759-1.415)	0.821	1.007 (0.736-1.378)	0.965	1.007 (0.725-1.397)	0.969
Class 3: Moderate-stable	1534	353	0.950 (0.837-1.079)	0.429	0.946 (0.833-1.076)	0.399	0.928 (0.811-1.063)	0.283
LDL-C								
Class 1: Stable	4651	1075	Reference		Reference		Reference	
Class 2: U-shape	222	41	0.831 (0.608-1.135)	0.244	0.867 (0.634-1.186)	0.372	0.848 (0.612-1.175)	0.322
Class 3: Inverse U-shape	88	20	0.761 (0.488-1.188)	0.230	0.790 (0.506-1.233)	0.299	0.796 (0.504-1.258)	0.329
HDL-C								
Class 1: Stable	4105	946	Reference		Reference		Reference	
Class 2: Inverse U-shape	177	53	1.204 (0.913-1.588)	0.189	1.228 (0.929-1.624)	0.150	1.277 (0.961-1.696)	0.091
Class 3: U-shape	679	137	0.931 (0.778-1.114)	0.434	0.960 (0.798-1.154)	0.662	0.955 (0.788-1.158)	0.639
**Age > 65 years**	1754	185						
TC								
Class 1: Moderate-stable	1651	174	Reference		Reference		Reference	
Class 2: Inverse U-shape	43	4	0.798 (0.296-2.152)	0.655	0.762 (0.282-2.058)	0.591	0.824 (0.304-2.236)	0.704
Class 3: U-shape	60	7	0.958 (0.450-2.041)	0.912	0.866 (0.406-1.849)	0.710	0.922 (0.431-1.974)	0.835
TG								
Class 1: Low-stable	1246	127	Reference		Reference		Reference	
Class 2: Elevated-increasing	28	4	1.964 (0.724-5.331)	0.185	1.677 (0.616-4.571)	0.312	1.806 (0.659-4.953)	0.251
Class 3: Moderate-stable	480	54	1.035 (0.752-1.425)	0.831	0.946 (0.684-1.309)	0.739	1.005 (0.721-1.401)	0.976
LDL-C								
Class 1: Stable	1642	172	Reference		Reference		Reference	
Class 2: U-shape	81	10	1.003 (0.529-1.899)	0.994	0.931 (0.490-1.767)	0.826	0.998 (0.524-1.899)	0.995

Class 3: Inverse U-shape	31	3	0.595 (0.190-1.868)	0.374	0.594 (0.189-1.864)	0.372	0.637 (0.202-2.005)	0.441
HDL-C								
Class 1: Stable	1421	148	Reference		Reference		Reference	
Class 2: Inverse U-shape	71	8	1.129 (0.553-2.303)	0.739	1.069 (0.521-2.192)	0.855	0.887 (0.387-2.031)	0.776
Class 3: U-shape	262	29	**1.524 (1.017-2.284)**	**0.041**	1.475 (0.976-2.229)	0.065	1.480 (0.971-2.256)	0.069

Bold values denote statistically significant. Model 1: Adjusted for age and male; Model 2: Adjusted for age, male, BMI, hypertension, and diabetes; Abbreviation: CAS, carotid atherosclerosis progression; HR, hazard ratio; TC, total cholesterol; TG, Triglyceride; LDL-C, low-density lipoprotein cholesterol; HDL-C, high-density lipoprotein cholesterol
